# Supplementary material for: Social familiarity improves fast-start escape performance in schooling fish
Source: Commun Biol. 2021 Jul 20;4:897. doi: 10.1038/s42003-021-02407-4 (PMC8292327; doi:10.1038/s42003-021-02407-4)
Supplement: Supplementary file 2 — Supplementary material [file 42003_2021_2407_MOESM2_ESM.pdf]

Supplementary material for "Social familiarity improves fast-start escape performance in schooling fish"

Lauren E. Nadler[1,2,3,*], Mark I. McCormick[1], Jacob L. Johansen[4], Paolo Domenici[5]

[1] ARC Centre of Excellence for Coral Reef Studies, James Cook University, Townsville, Queensland, 4811, Australia

[2] College of Science and Engineering, James Cook University, Townsville, Queensland, 4811 Australia

[3] Current address: Department of Marine and Environmental Sciences, Nova Southeastern University, Dania Beach, FL 33004, USA

[4] Hawai'i Institute of Marine Biology, University of Hawai'i at Manoa, Kaneohe, HI, 96744, USA

[5] CNR-IBF, Institute of Biophysics, Pisa, Italy

[*] Email: lnadler@nova.edu

Table of Contents

1. SUPPLEMENTARY FIGURES 3

FIG. S1. TIME TO FAMILIARITY IN THE CORAL REEF DAMSELFISH *CHROMIS VIRIDIS*. 3
FIG. S3. THE FAST-START ESCAPE RESPONSE OF FAMILIAR VERSUS UNFAMILIAR SCHOOLS OF THE DAMSELFISH *CHROMIS VIRIDIS* AT VARYING DISTANCES FROM THE STIMULUS 4
FIG. S4. FOREST PLOTS SHOWING THE EFFECT SIZES (I.E. STRUCTURE CORRELATION COEFFICIENT) AND THEIR 95% CONFIDENCE INTERVALS GRAPHICALLY FOR FIXED EFFECTS (AND THEIR INTERACTIONS) IN ALL MIXED-EFFECTS MODELS..... 5

2. SUPPLEMENTARY TABLES 14

3. SUPPLEMENTARY REFERENCES 19

1. Supplementary figures

Fig. S1. Time to familiarity in the coral reef damselfish *Chromis viridis*. White bars indicate the count of individuals (out of eight total per day) that preferred the familiar school, while the grey bars denote those individuals that preferred the unfamiliar school. Preference was defined as the school that the focal individual spent the greatest proportion of time with during the 15-min trial. The dotted line denotes the null hypothesis of an even 50:50 split between the two schools on each day (which would indicate no preference). Duration of exposure to school-mates had a significant effect on whether individual fish would choose schools of known (i.e., familiar) or unknown (i.e., unfamiliar) individuals (GLM: $\chi^2 = 4.52$, $p = 0.033$) and no differences were discovered among schools (GLM: $\chi^2 = 0.33$, $p = 0.563$). The majority of individuals first began exhibiting a preference for familiar individuals on day 15 (63%). This trend continued through days 17 (75%), 19 (88%) and 21 (75%), indicating that familiarity is established within three weeks.

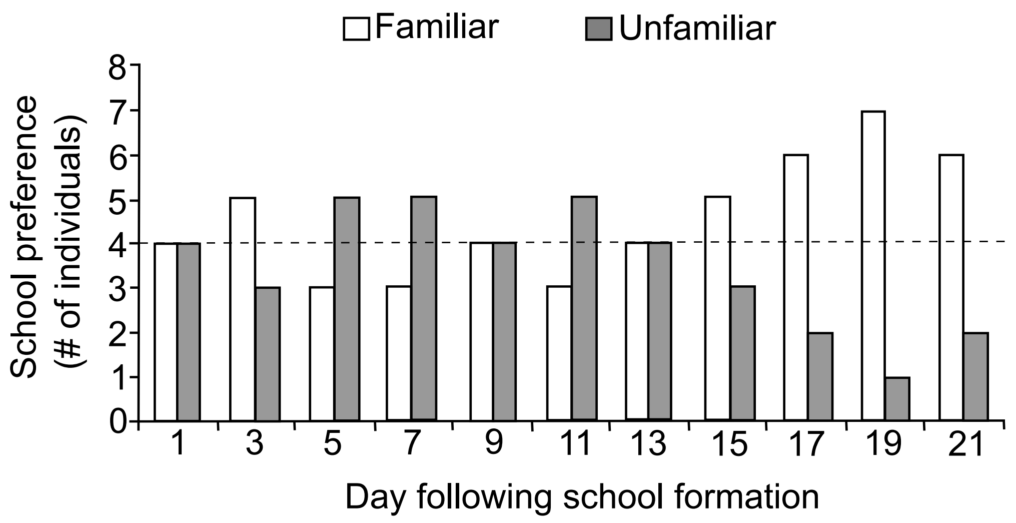

Fig. S3. The fast-start escape response of familiar versus unfamiliar schools of the damselfish *Chromis viridis* at varying distances from the stimulus (i.e., stimulus distance), including (**a**) average turning rate and (**b**) distance covered.

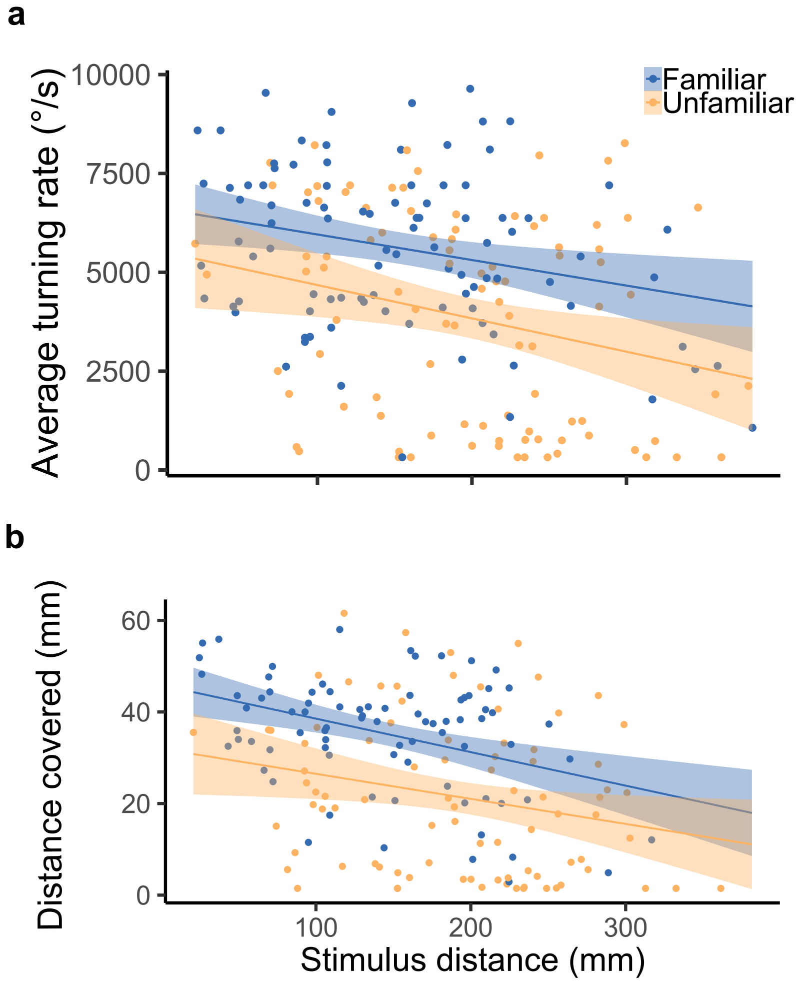

Fig. S4. Forest plots showing the effect sizes (i.e. structure correlation coefficient) and their 95% confidence intervals graphically for fixed effects (and their interactions) in all mixed-effects models. Plots below illustrate models assessing latency (a, subsequent responders; b, proportion of fast & slow responses, categorized by latencies < 16ms and > 16ms, respectively[1]), average turning rate (c, first responders; d, subsequent responders), distance covered (e, first responders; f, subsequent responders), nearest neighbor distance (g, all responders), school alignment (h, all responders), and school area (i).

A) Latency (subsequent responders)

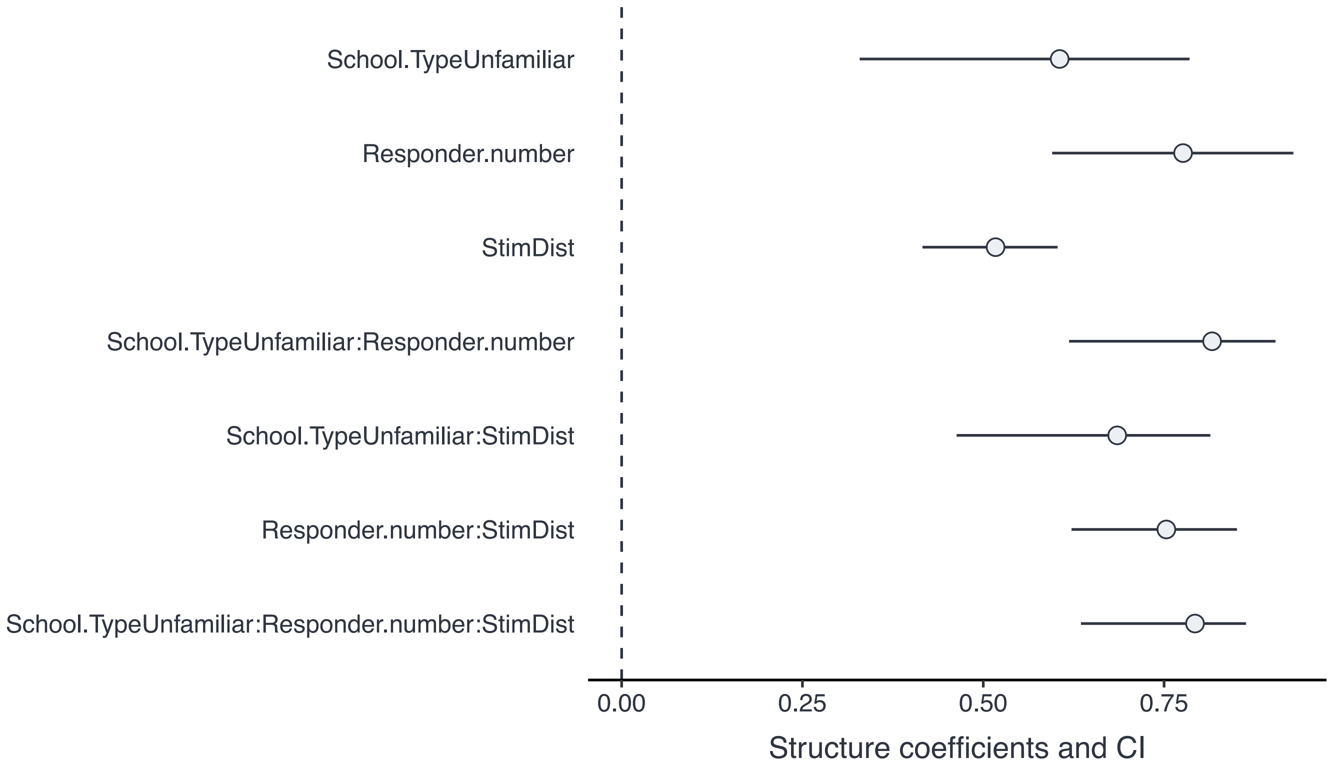

B) Latency (proportion of fast vs. slow responses)

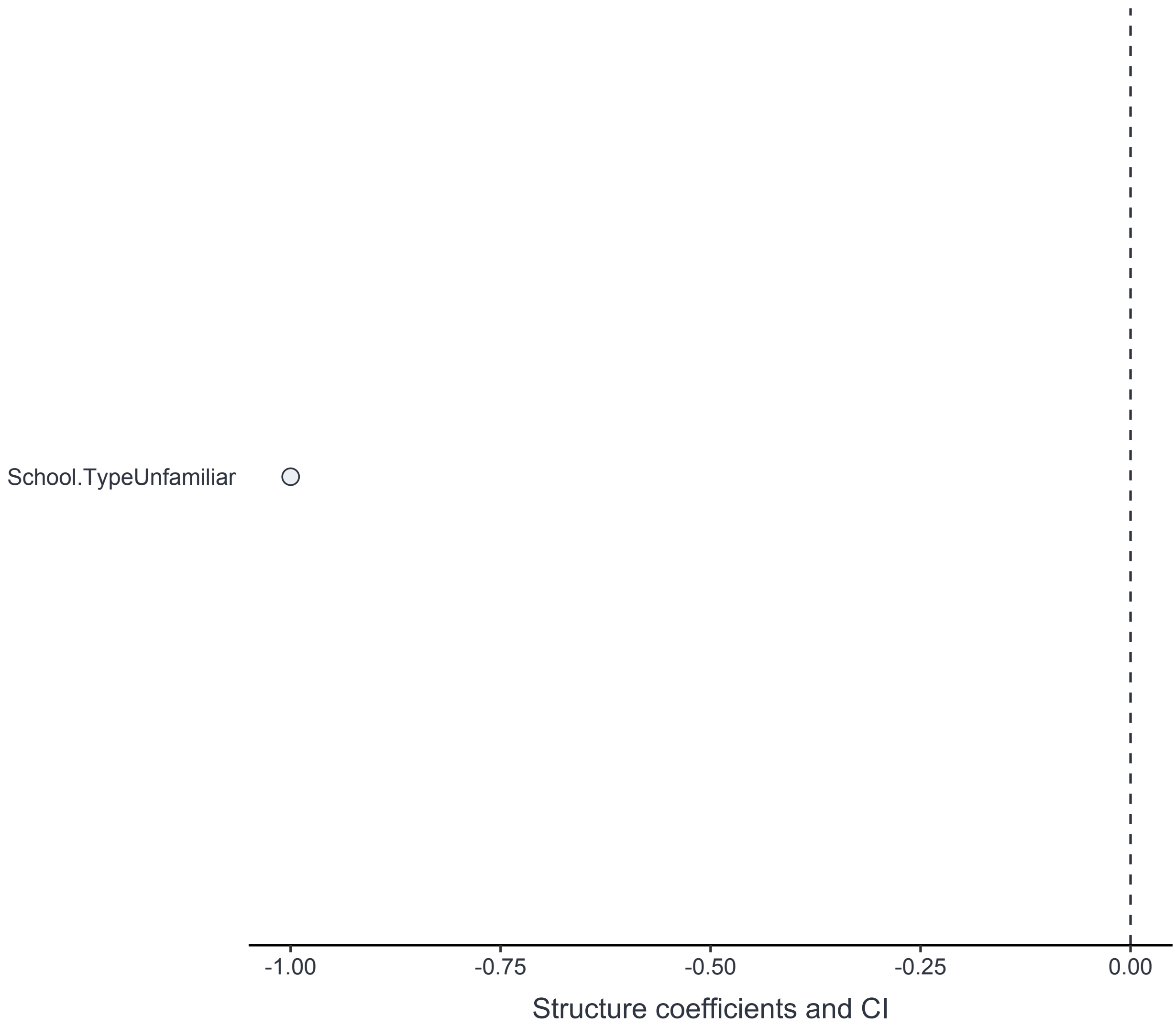

C) Average turning rate (first responders)

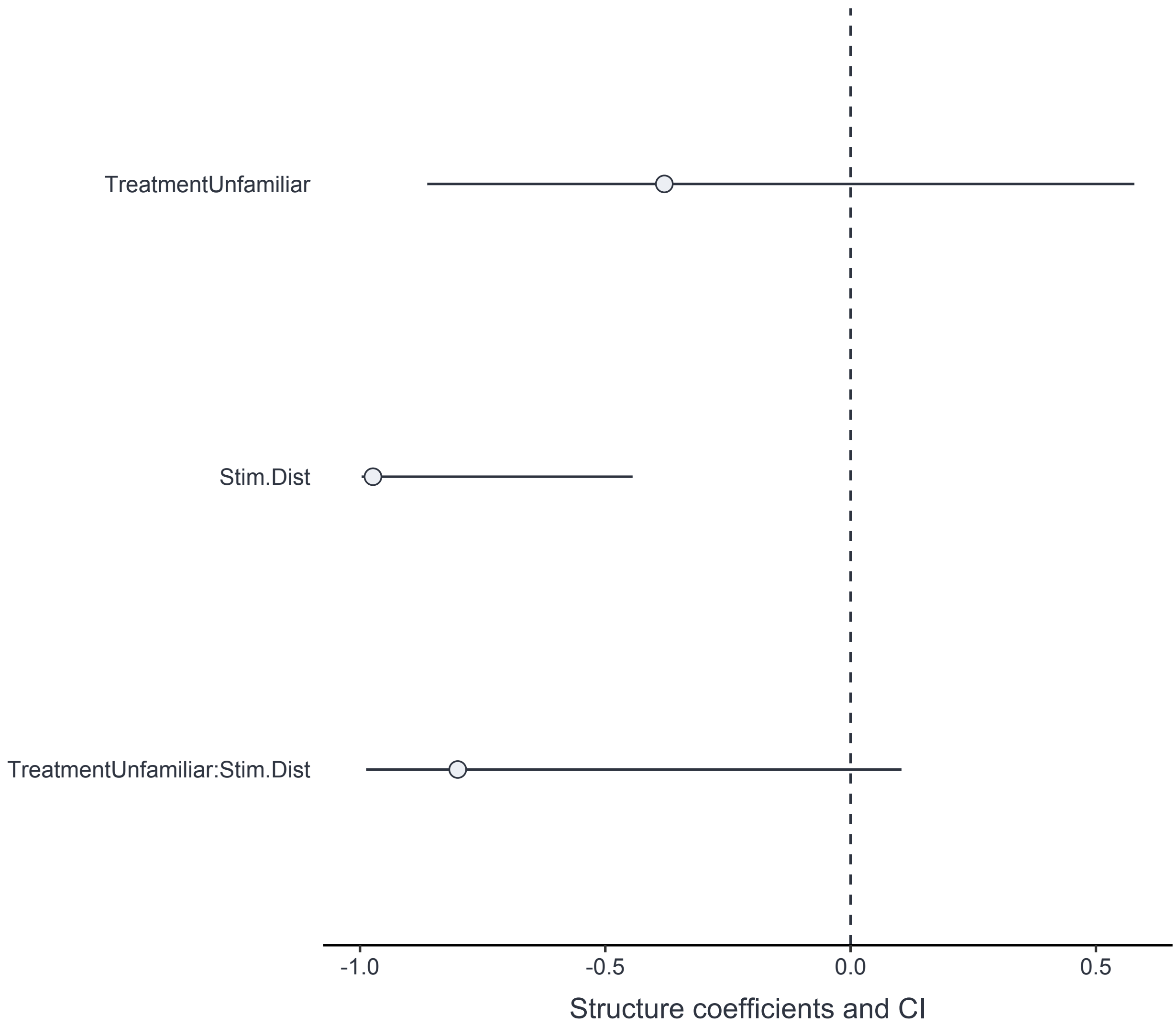

D) Average turning rate (subsequent responders)

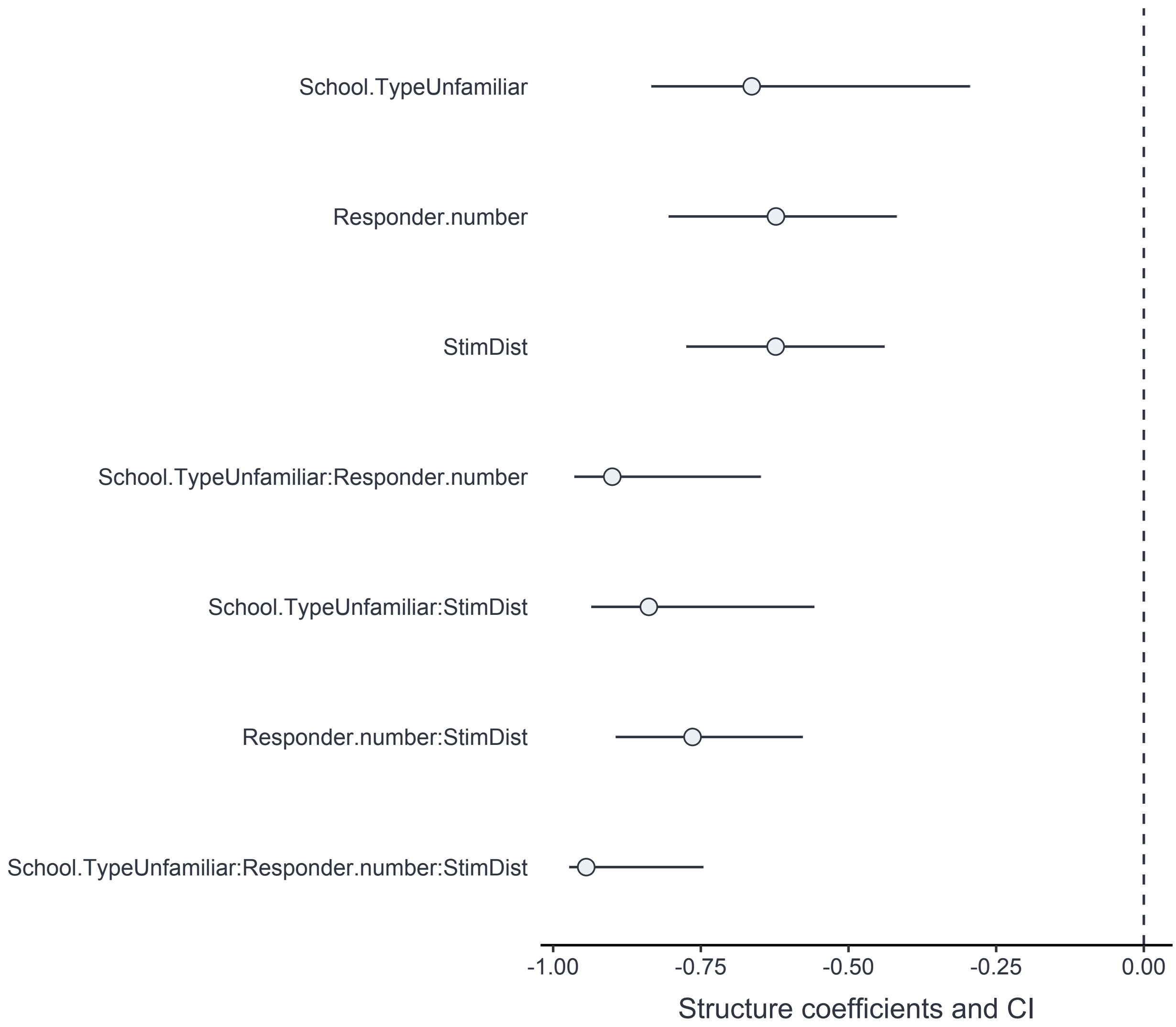

E) Distance covered (first responders)

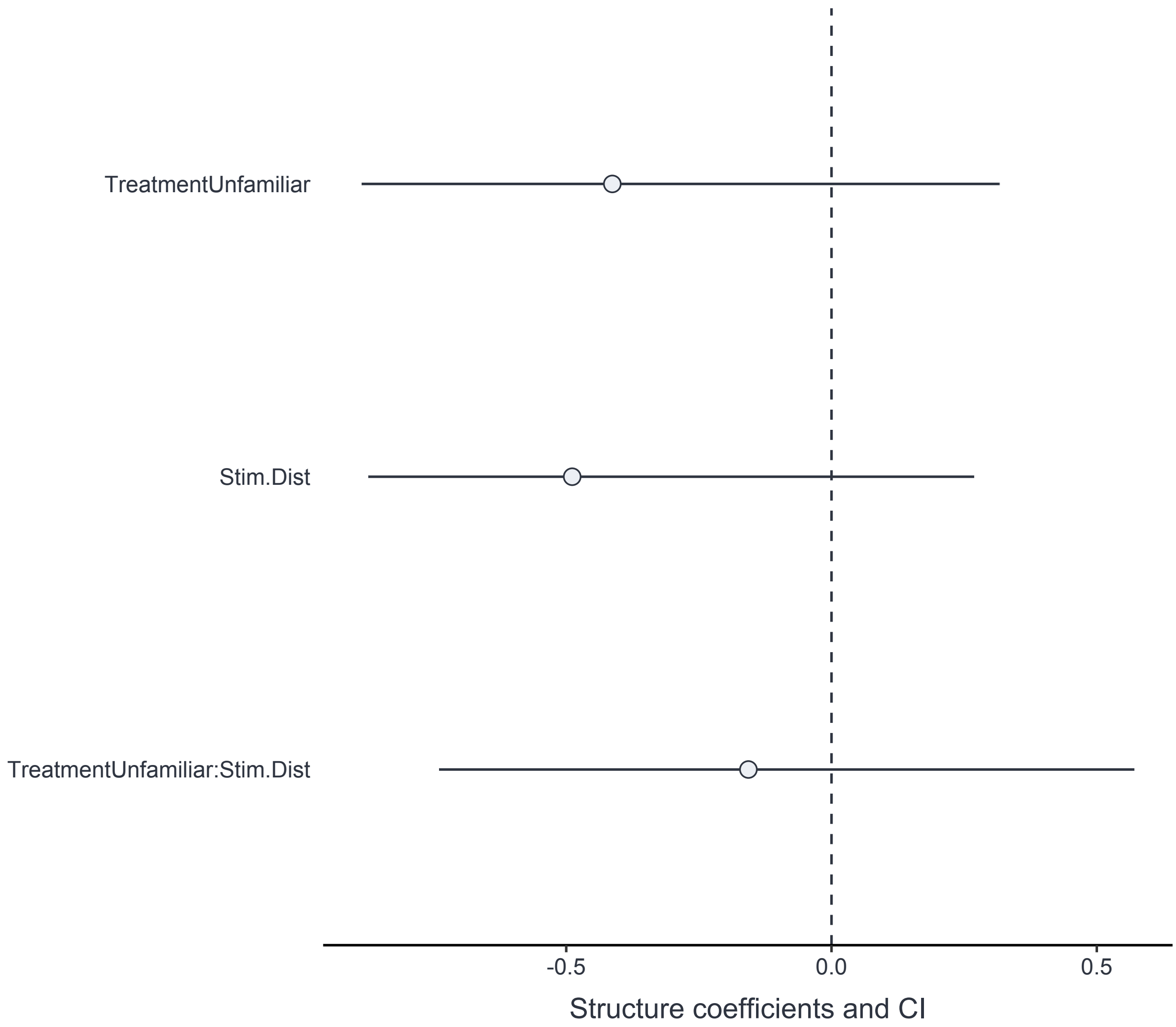

F) Distance covered (subsequent responders)

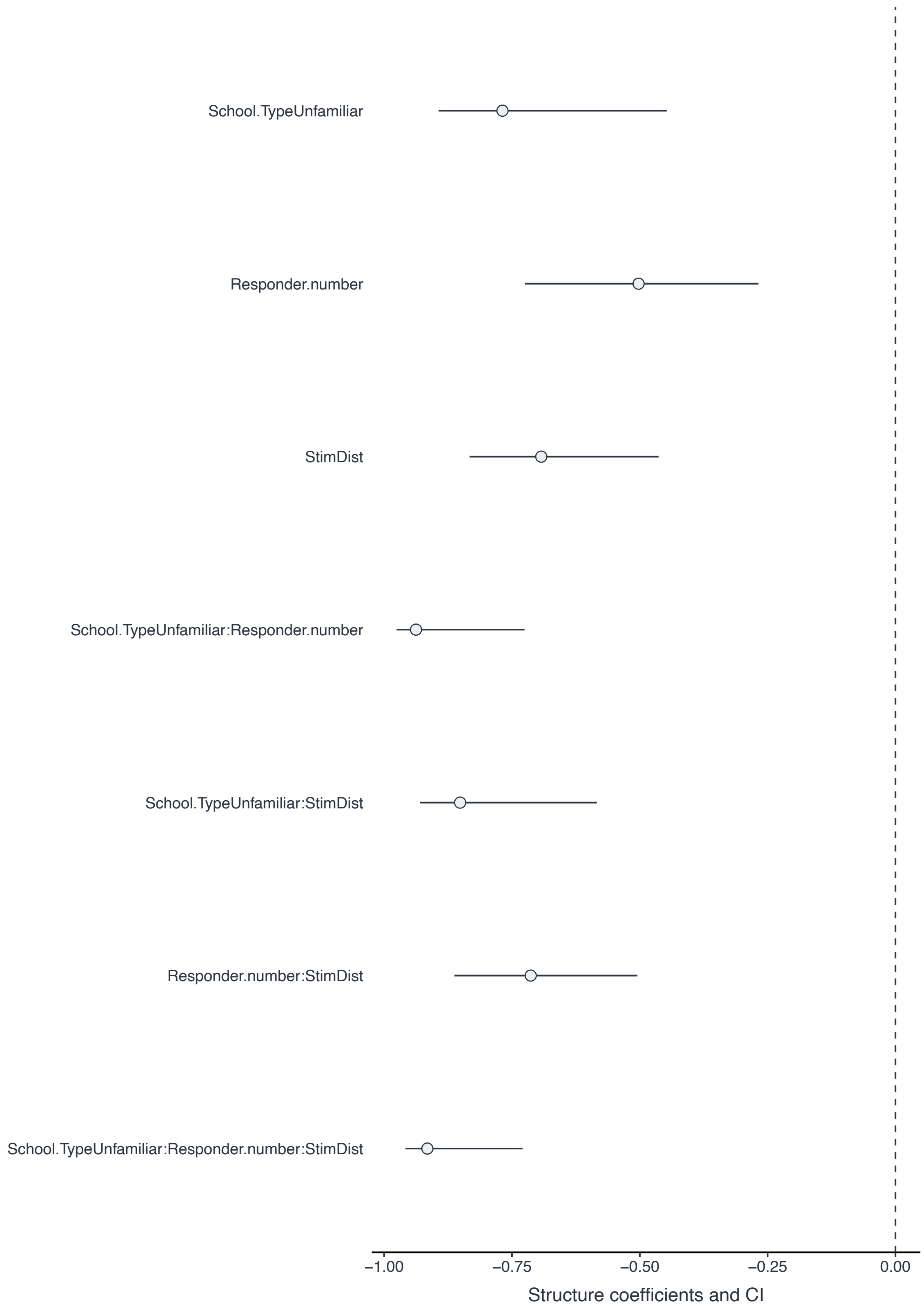

G) Nearest neighbor distance (all responders)

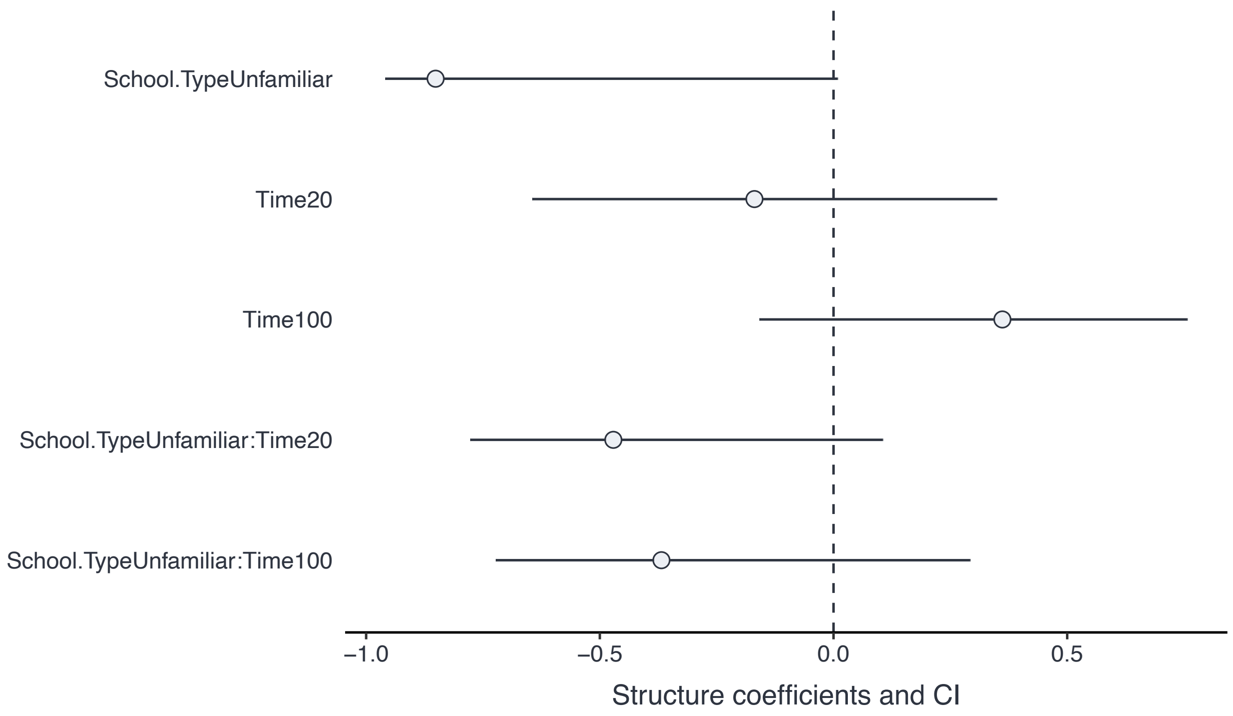

H) School alignment (all responders)

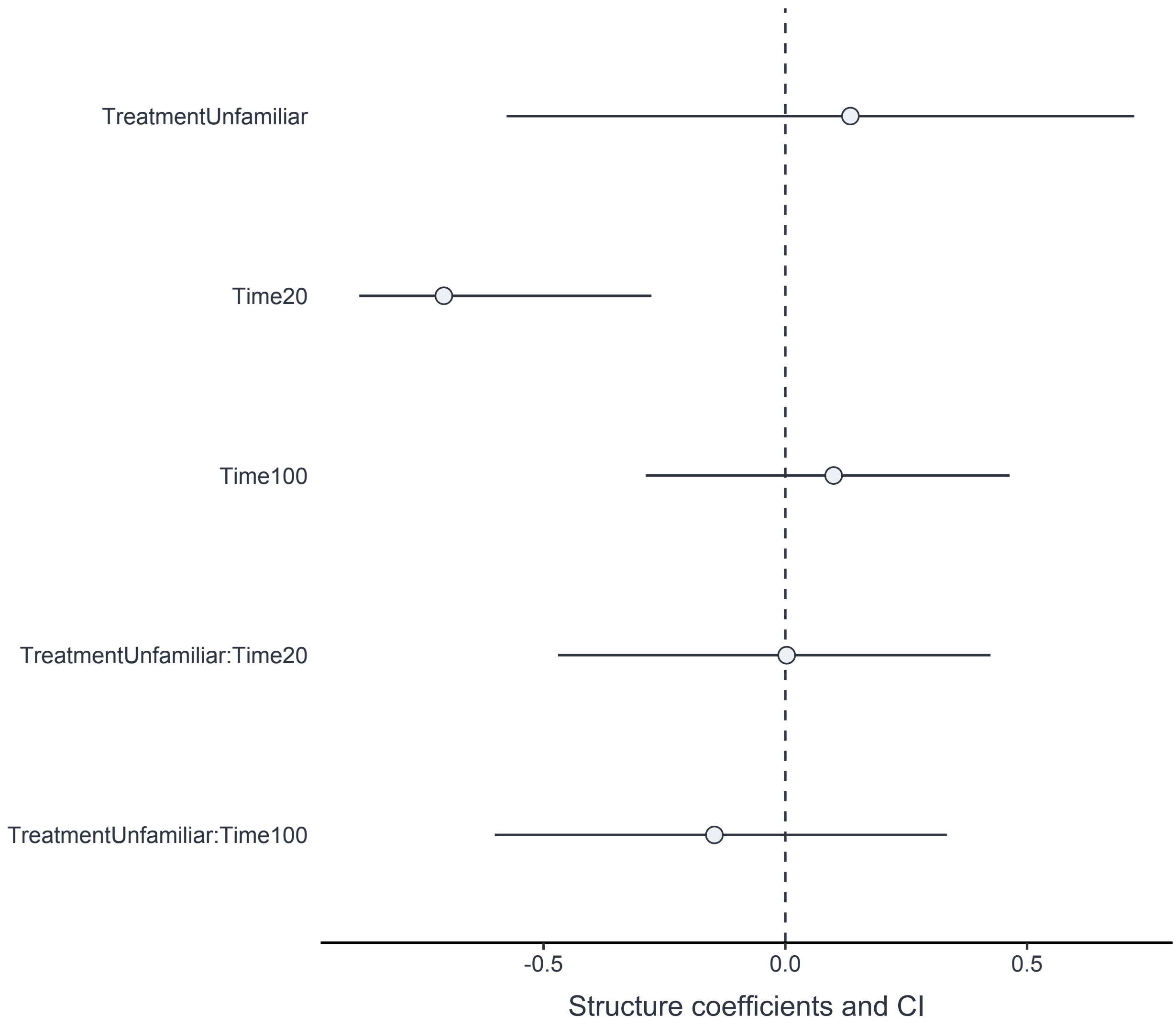

I) School area

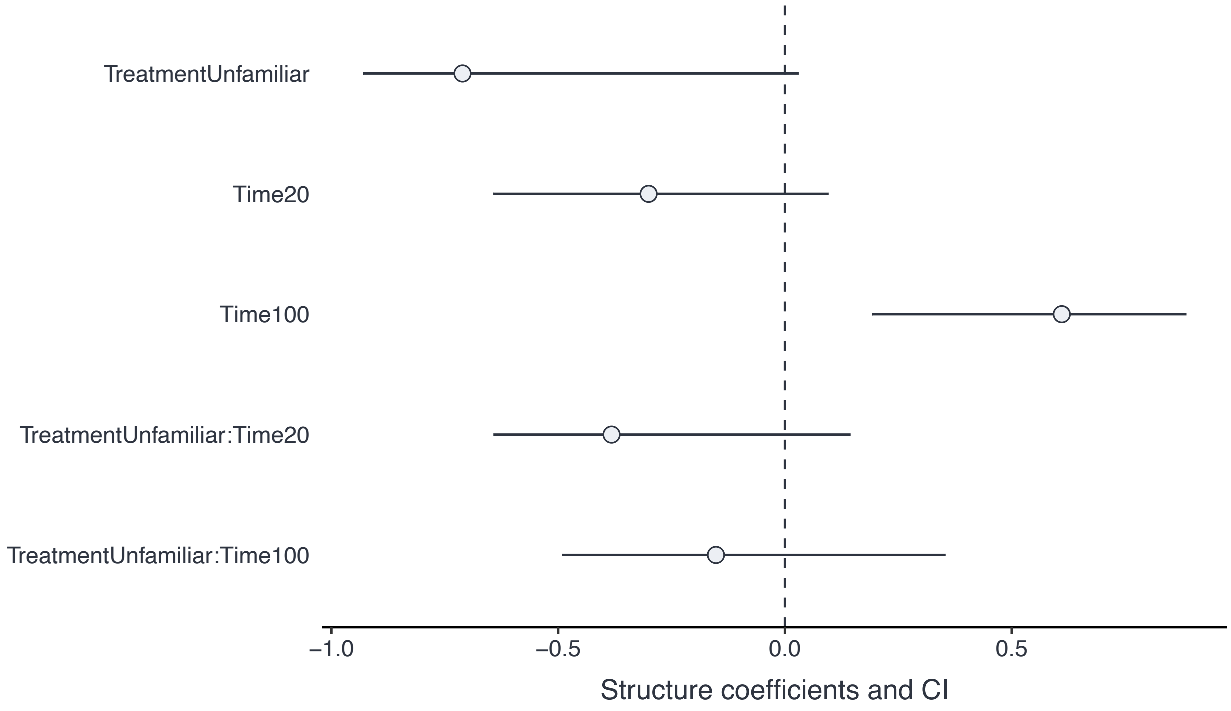

2. Supplementary tables

Table S1. Sample sizes for responder numbers across all experimental schools by familiarity treatment (n = 24 schools composed of 8 fish each, split evenly across familiarity treatments). Ties occurred in 13 of the tested schools (number of ties: 2-4 individuals per school; number of schools exhibiting ties by treatment: n=9 familiar schools, n=4 unfamiliar schools).

	**1**	**2**	**3**	**4**	**5**	**6**	**7**	**8**
**Familiar**	25	5	12	13	11	7	11	12
**Unfamiliar**	17	10	11	12	10	12	13	11

Table S2. Effect tests assessing variation in the role of familiarity (familiar versus unfamiliar) in the latency of responses in (**a**) first responders (responder number 1) and (**b**) subsequent responders (responder numbers 2 – 8), using generalized linear model analysis and linear-mixed effects model analysis, respectively, in schools of the blue-green Chromis (*Chromis viridis*) composed of eight fish each (n = 12 familiar and n = 12 unfamiliar schools) following a simulated predator attack. Stimulus distance was included in all models, which indicates the distance from the stimulus to each fish´s center of mass (in mm).

(A)

**Factor**	**d.f.**	**F-value**	**p-value**	**R^2**
Familiarity	1,22	9.15	0.007	0.43
Stimulus distance	1,21	23.37	0.0001	
Familiarity*Stimulus distance	1,20	0.34	0.57	

(B)

**Factor**	**d.f.**	**F-value**	**p-value**	**R^2m, R^2c**
Familiarity	1,22	12.26	0.002	0.51, 0.86
Responder number	1,125	207.88	<0.0001	
Stimulus distance	1,131	0.68	0.41	
Familiarity*Responder number	1,124	0.27	0.60	
Familiarity*Stimulus Distance	1,131	0.64	0.43	
Responder number*Stimulus distance	1,123	0.06	0.81	
Familiarity*Responder number*Stimulus distance	1,123	0.79	0.38	

Table S3. Effect tests assessing variation in the role of familiarity (familiar versus unfamiliar) in the average turning rate (in °/s) of (**a**) first responders (responder number 1) and (**b**) subsequent responders (responder numbers 2 – 8), using linear-mixed effects model analysis, in schools of the blue-green Chromis (*Chromis viridis*) composed of eight fish each (n = 12 familiar and n = 12 unfamiliar schools) following a simulated predator attack. Stimulus distance was included in all models, which indicates the distance from the stimulus to each fish´s center of mass (in mm).

(A)

**Factor**	**d.f.**	**F-value**	**p-value**	**R^2m, R^2c**
Familiarity	1,21	0.08	0.78	0.15, 0.66
Stimulus distance	1,34	7.36	0.01	
Familiarity*Stimulus distance	1,34	0.28	0.60	

(B)

**Factor**	**d.f.**	**F-value**	**p-value**	**R^2m, R^2c**
Familiarity	1,22	7.26	0.01	0.29, 0.58
Responder number	1,131	21.60	<0.0001	
Stimulus distance	1,142	3.35	0.07	
Familiarity*Responder number	1,131	0.42	0.52	
Familiarity*Stimulus Distance	1,141	0.07	0.79	
Responder number*Stimulus distance	1,128	0.62	0.43	
Familiarity*Responder number*Stimulus distance	1,127	0.54	0.46	

Table S4. Effect tests assessing variation in the role of familiarity (familiar versus unfamiliar) in the distance covered (i.e., the distance moved during the first 42 ms of the reaction, in mm) by (**a**) first responders (responder number 1) and (**b**) subsequent responders (responder numbers 2 – 8), using linear-mixed effects model analysis, in schools of the blue-green chromis (*Chromis viridis*) composed of eight fish each (n = 12 familiar and n = 12 unfamiliar schools) following a simulated predator attack. Stimulus distance was included in all models, which indicates the distance from the stimulus to each fish´s center of mass (in mm).

(A)

**Factor**	**d.f.**	**F-value**	**p-value**	**R^2m, R^2c**
Familiarity	1,20	1.00	0.33	0.22, 0.58
Stimulus distance	1,28	0.94	0.34	
Familiarity*Stimulus distance	1,30	5.96	0.02	

(B)

**Factor**	**d.f.**	**F-value**	**p-value**	**R^2m, R^2c**
Familiarity	1,23	9.34	0.006	0.31, 0.57
Responder number	1,115	10.35	0.002	
Stimulus distance	1,122	3.72	0.06	
Familiarity*Responder number	1,114	5.09	0.03	
Familiarity*Stimulus Distance	1,123	0.98	0.32	
Responder number*Stimulus distance	1,113	0.50	0.48	
Familiarity*Responder number*Stimulus distance	1,114	0.17	0.69	

Table S5. Effect of familiarity on escape performance of fish schools (*Chromis viridis*) over time following a simulated predator attack (0, 20 and 100 ms post-stimulation), including (**a**) nearest neighbor distance (denotes the distance to the closest neighbor, in mm), (**b**) school area (indicates the school's horizontal spread, in cm^2), and (**c**) alignment (a measure of the variation in the orientation of all school members, characterized by the length of mean circular vector, i.e., r), as assessed using linear mixed-effects model analysis.

(A)

**Factor**	**d.f.**	**F-value**	**p-value**	**R^2m, R^2c**
Familiarity	1,22	3.83	0.06	0.03, 0.10
Time	2,548	1.05	0.35	
Familiarity*Time	2,548	1.16	0.31	

(B)

**Factor**	**d.f.**	**F-value**	**p-value**	**R^2m, R^2c**
Familiarity	1,22	3.20	0.09	0.15, 0.59
Time	2,44	5.03	0.01	
Familiarity*Time	2,44	1.64	0.20	

(C)

**Factor**	**d.f.**	**F-value**	**p-value**	**R^2m, R^2c**
Familiarity	1,22	0.09	0.76	0.12, 0.54
Time	2,44	5.47	0.008	
Familiarity*Time	2,44	3.73	0.03	

3. Supplementary references

1 Eaton, R. C., Lavender, W. A. & Wieland, C. M. Identification of Mauthner-initiated response patterns in goldfish: Evidence from simultaneous cinematography and electrophysiology. *J. Comp. Phys. A* **144**, 521-531 (1981).
