# Supplementary material for: Social familiarity improves fast-start escape performance in schooling fish
Source: Commun Biol. 2021 Jul 20;4:897. doi: 10.1038/s42003-021-02407-4 (PMC8292327; doi:10.1038/s42003-021-02407-4)
Supplement: Supplementary file 3 — Reporting Summary [file 42003_2021_2407_MOESM3_ESM.pdf]

nature research

Corresponding author(s): Lauren Nadler

Last updated by author(s): Jun 18, 2021

Reporting Summary

Nature Research wishes to improve the reproducibility of the work that we publish. This form provides structure for consistency and transparency in reporting. For further information on Nature Research policies, see our Editorial Policies and the Editorial Policy Checklist.

Statistics

For all statistical analyses, confirm that the following items are present in the figure legend, table legend, main text, or Methods section.

n/a	Confirmed	
☐	☒	The exact sample size (*n*) for each experimental group/condition, given as a discrete number and unit of measurement
☐	☒	A statement on whether measurements were taken from distinct samples or whether the same sample was measured repeatedly
☐	☒	The statistical test(s) used AND whether they are one- or two-sided<br>*Only common tests should be described solely by name; describe more complex techniques in the Methods section.*
☐	☒	A description of all covariates tested
☐	☒	A description of any assumptions or corrections, such as tests of normality and adjustment for multiple comparisons
☐	☒	A full description of the statistical parameters including central tendency (e.g. means) or other basic estimates (e.g. regression coefficient) AND variation (e.g. standard deviation) or associated estimates of uncertainty (e.g. confidence intervals)
☐	☒	For null hypothesis testing, the test statistic (e.g. *F*, *t*, *r*) with confidence intervals, effect sizes, degrees of freedom and *P* value noted<br>*Give P values as exact values whenever suitable.*
☒	☐	For Bayesian analysis, information on the choice of priors and Markov chain Monte Carlo settings
☐	☒	For hierarchical and complex designs, identification of the appropriate level for tests and full reporting of outcomes
☐	☒	Estimates of effect sizes (e.g. Cohen's *d*, Pearson's *r*), indicating how they were calculated

Our web collection on statistics for biologists contains articles on many of the points above.

Software and code

Policy information about availability of computer code

Data collection	Video recorded trials of fish behaviour were analysed manually and blinded using the ImageJ software.
Data analysis	All statistical analysis was conducted in the R Statistical Environment (v3.2.4).

For manuscripts utilizing custom algorithms or software that are central to the research but not yet described in published literature, software must be made available to editors and reviewers. We strongly encourage code deposition in a community repository (e.g. GitHub). See the Nature Research guidelines for submitting code & software for further information.

Data

Policy information about availability of data

All manuscripts must include a data availability statement. This statement should provide the following information, where applicable:

- Accession codes, unique identifiers, or web links for publicly available datasets
- A list of figures that have associated raw data
- A description of any restrictions on data availability

All data, code, and an annotated pdf document of the statistical analysis (R Markdown file) are available through the NSUWorks Data Repository (https://nsuworks.nova.edu/occ_facdatasets/13/). Raw data is illustrated in the dotplots on graphs that contain error bars (Figures 2c, 3a, 4 and 5).

Field-specific reporting

Ecological, evolutionary & environmental sciences study design

All studies must disclose on these points even when the disclosure is negative.

Study description	Using social groups (i.e. schools) of the tropical damselfish Chromis viridis (Pomacentridae), we examined how familiarity influences fast-start escape responses, the primary defensive behaviour in a wide range of taxa (e.g., fish, sharks, and larval amphibians) that is controlled by a network of reticulospinal neurons in response to a threat. For each individual, we focused on reactivity performance (i.e., latency to react) and kinematic performance (i.e., agility and propulsive performance) of the response to a simulated predator attack, while distinguishing between "first responders" (i.e., direct response to stimulation) and "subsequent responders" (i.e., followers whose response is triggered by integrated direct and social stimulation).
Research sample	This experiment was conducted at the Lizard Island Research Station (LIRS) in the northern Great Barrier Reef, Australia (14°40′08″S; 145°27′34″E). Distinct schools of the tropical damselfish C. viridis (Pomacentridae, standard length: 3.33 + 0.02 cm, mean + s.e.; n = 192 fish) were collected from different reefs (separated by 400-3000m) in the lagoon adjacent to LIRS using hand nets, a dilute anaesthetic solution of clove oil and barrier nets. Once collected, all wild schools were maintained in sensory isolation from one another (both visual and olfactory isolation) in a flow-through aquaria system at a density of approximately 1 fish per 2.5L. Fish were fed to satiation twice daily with INVE Aquaculture pellets and newly hatched Artemia spp.
Sampling strategy	Sample size was maximized according to availability of distinct social groups on the reefs of Lizard Island that were accessible to the researchers and based on the time available in the field to conduct these studies.
Data collection	All experimental trails were video recorded and later analyzed blind to treatment by the first author.
Timing and spatial scale	All experimental procedures were conducted in the Austral summer 2013 (October to December), with video analysis completed in 2014.
Data exclusions	No data were excluded.
Reproducibility	These trials were conducted using a variety of fish schools over two months at the Lizard Island Research Station in 2013, in order to ensure that the results couldn´t be driven by unique factors to a limited number of fishes. The entirety of the experiment has not been repeated, in part due to habitat degradation at the study site (through coral bleaching and cyclone damage at the study site from 2014 to 2020).
Randomization	Individuals were assigned systematically to fish schools following collection from the field, in order to ensure that there was a consistent size variation within groups and across familiarity treatments. Group number was included as a random effect in all models examining individual traits.
Blinding	All video analysis was completed blind. The first author completed all video analysis, following blinding of video labels by a researcher acknowledged in the Acknowledgments section, in order to prevent the possibility of unconscious bias in the results.

Did the study involve field work? ☒ Yes ☐ No

Field work, collection and transport

Field conditions	All fish were collected in the Austral summer 2013 when ambient temperatures ranged from 27-29 degrees Celsius.
Location	All live animal experimentation occurred at the Lizard Island Research Station in the northern Great Barrier Reef, Australia (14°40′08″ S; 145°27′34″E) in the Austral summer 2013, with animals collected from shallow water reefs (< 4m depth).
Access & import/export	Animals were collected under the Great Barrier Reef Marine Park Authority permit G13/35909.1 and General Fisheries Permit number 170251.
Disturbance	All fish were returned to the reef following experimentation in order to minimize disturbance to local reef fish populations.

Reporting for specific materials, systems and methods

We require information from authors about some types of materials, experimental systems and methods used in many studies. Here, indicate whether each material, system or method listed is relevant to your study. If you are not sure if a list item applies to your research, read the appropriate section before selecting a response.

Materials & experimental systems

n/a	Involved in the study
☒	☐ Antibodies
☒	☐ Eukaryotic cell lines
☒	☐ Palaeontology and archaeology
☐	☒ Animals and other organisms
☒	☐ Human research participants
☒	☐ Clinical data
☒	☐ Dual use research of concern

Methods

n/a	Involved in the study
☒	☐ ChIP-seq
☒	☐ Flow cytometry
☒	☐ MRI-based neuroimaging

Animals and other organisms

Policy information about studies involving animals; ARRIVE guidelines recommended for reporting animal research

Laboratory animals	Distinct schools of the tropical damselfish Chromis viridis were collected from the reef for this study.
Wild animals	Schools of the damselfish Chromis viridis were collected using hand nets, a dilute anaesthetic solution of clove oil, and barrier nets.
Field-collected samples	Once collected, all wild schools were maintained in sensory isolation from one another (both visual and olfactory isolation) in a flow-through aquaria system at a density of approximately 1 fish per 2.5L. Fish were fed to satiation twice daily with INVE Aquaculture pellets and newly hatched Artemia spp.
Ethics oversight	This research was conducted following guidelines and regulations from the James Cook University Animal Ethics Committee (permit number A2103).

Note that full information on the approval of the study protocol must also be provided in the manuscript.
